# Albumin fibrillization induces apoptosis via integrin/FAK/Akt pathway

**DOI:** 10.1186/1472-6750-9-2

**Published:** 2009-01-08

**Authors:** Chun-Yung Huang, Chi-Ming Liang, Chiao-Li Chu, Shu-Mei Liang

**Affiliations:** 1 Institute of Biotechnology, National Cheng Kung University, Tainan, Taiwan; 2 Institute of Cellular and System Medicine, National Health Research Institutes, Miaoli County, Taiwan; 3 Institute of Biological Chemistry, Academia Sinica, Taipei, Taiwan; 4 Agricultural Biotechnology Research Center, Academia Sinica, Taipei, Taiwan

## Abstract

**Background:**

Numerous proteins can be converted to amyloid-like fibrils to increase cytotoxicity and induce apoptosis, but the methods generally require a high concentration of protein, vigorous shaking, or fibril seed. As well, the detailed mechanism of the cytotoxic effects is not well characterized. In this study, we have developed a novel process to convert native proteins into the fibrillar form. We used globular bovine serum albumin (BSA) as a model protein to verify the properties of the fibrillar protein, investigated its cellular effects and studied the signaling cascade induced by the fibrillar protein.

**Results:**

We induced BSA, a non-cytotoxic globular protein, to become fibril by a novel process involving Superdex-200 column chromatography in the presence of anionic or zwittergenic detergent(s). The column pore size was more important than column matrix composite in fibril formation. The fibrillar BSA induced apoptosis in BHK-21 cell as well as breast cancer cell line T47D. Pre-treating cells with anti-integrin antibodies blocked the apoptotic effect. Fibrillar BSA, but not globular BSA, bound to integrin, dephosphorylated focal adhesion kinase (FAK), Akt and glycogen synthase kinase-3β (GSK-3β).

**Conclusion:**

We report on a novel process for converting globular proteins into fibrillar form to cause apoptosis by modulating the integrin/FAK/Akt/GSK-3β/caspase-3 signaling pathway. Our findings may be useful for understanding the pathogenesis of amyloid-like fibrils and applicable for the development of better therapeutic agents that target the underlying mechanism(s) of the etiologic agents.

## Background

After glycation [1], sonication [2], or incubation at high temperature [3-7], numerous proteins can be converted to amyloid-like fibrils with common properties such as more notable β-structure, fibrillar morphology, protofilament substructure, cross-β diffraction pattern, and binding to Congo red or amyloid-specific dye thioflavin T (ThT) [8-12]. The formation of amyloid fibrils can be promoted by the presence of ethanol [7,13], sodium dodecyl sulfate (SDS) [14-16], or other anionic detergents [17]. However, these methods generally require a high concentration of protein, vigorous shaking, or the addition of fibril seed and are not easy to isolate fibrillar protein from others such as oligomers, protofibrils or non-fibrillar proteins that may also exist in the solution [1,2,7,15,16,18,19].

Amyloid fibrils are cytocytotoxic [20-23], however, it is still debatable as to whether oligomers, mature fibrils, protofibrils or target cellular types are the most critical factors in dictating cellular damage [24-27]. Study of disease-associated proteins such as the amyloid-β peptide (Aβ), α-synuclein, and transthyretin shows that common features of specific types of aggregates rather than specific amino acid sequences are positively related to cytotoxicity [28]. Amyloid oligomers from several different proteins have been shown to share the ability to permeabilize cellular membranes and lipid bilayers, which is thus proposed as the primary toxic mechanism of amyloid pathogenesis [26]. Lysozyme amyloid oligomers and fibrils, however, exert cytotoxicity by acting within different time-scales and via apoptosis-like and necrosis-like cell death, respectively [23]. Study with human islet amyloid polypeptide (hiAPP), the major constituent of islet amyloid, on the other hand, shows that growth of hiAPP fibrils at the cellular membrane, rather than pre-formed oligomers or fibrils are responsible for the observed membrane damage [27]. Recently, it has been demonstrated with specific integrin-blocking antibodies that amyloid-β peptide (Aβ) deposition and neurotoxicity in human cortical primary neurons are mediated through α2β1 and αVβ1 integrin receptors [29]. Whether binding of Aβ to integrin results in change of down stream signaling molecules such as Akt, Bad, forkhead transcription factors, glycogen synthase kinase-3 (GSK-3β), caspase-9 and eventually lead to apoptosis [30-35], however, has not been clarified.

Here we used a detergent-assisted refolding method [36] developed previously for the refolding of recombinant capsid protein VP1 of foot-and-mouth disease virus (FMDV) to promote the formation of fibrillar proteins in general. We chose a model protein, BSA, for evaluating fibrillation of protein using the new method. Furthermore, the fibrillar BSA processed by this new method was used to investigate the cellular effect and signaling cascade of amyloid-like fibrils. Our findings suggest that amyloid-like fibrillar BSA induced cellular cytotoxicity and apoptosis via modulating the integrin/focal adhesion kinase (FAK)/Akt/GSK-3β/caspase-3 pathway.

## Results

### Conversion of globular protein into fibril by column chromatography

**Figure 1:**
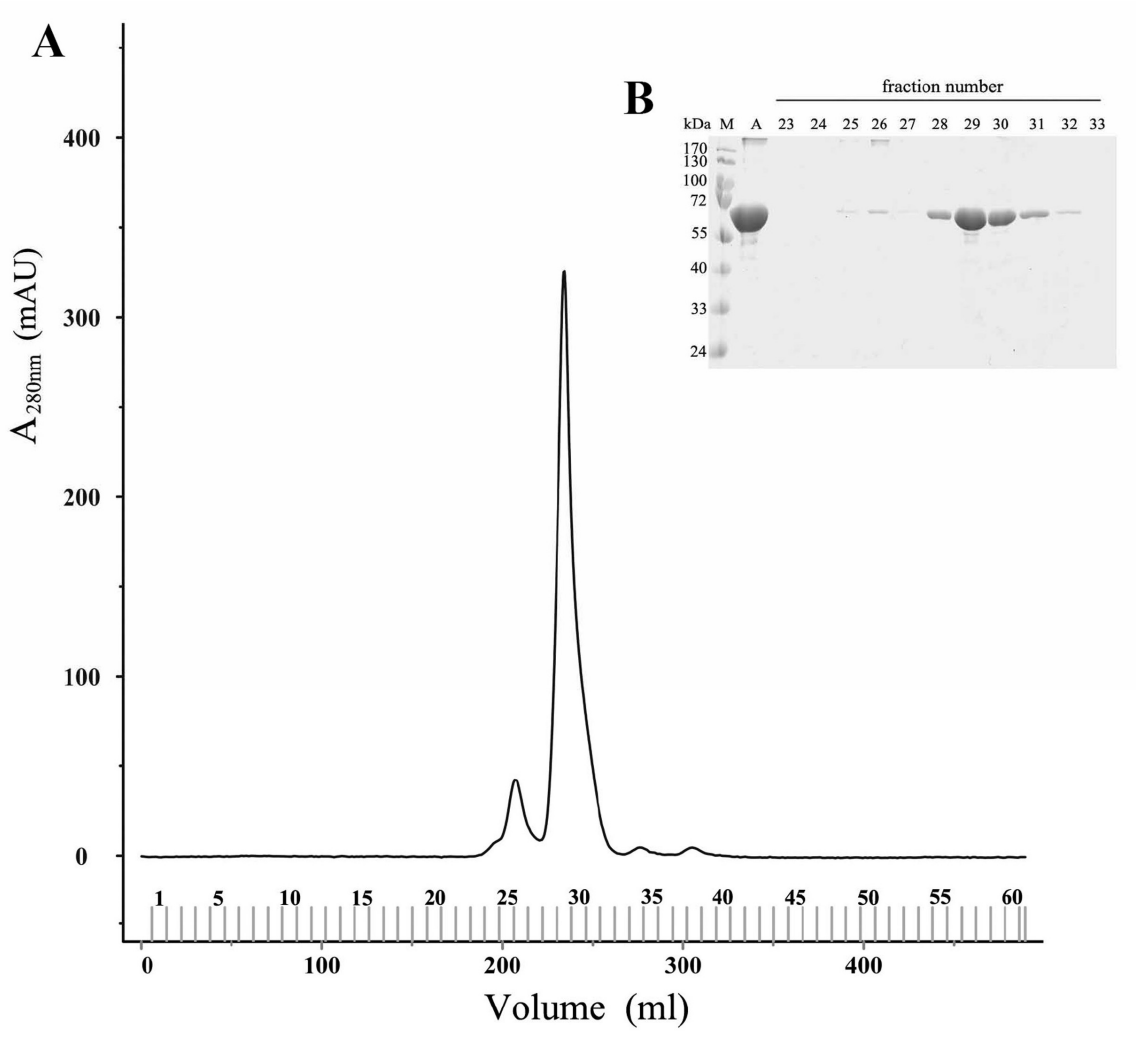
**Formation of fibrillar BSA on Superdex-200 column chromatography**. (A) BSA (2 mg/ml dissolved in PBS containing 1% SDS) was applied to a Superdex-200 column and eluted at the rate of 1 ml/min with a buffer solution containing 0.05% SDS. (B) Fractions from 23 to 33 were analyzed by SDS-PAGE and stained with Coomassie Blue. Lane A contains G-BSA as control. Before loading to the gel, the fibrillar BSA was solubilized in a sample buffer solution containing 1% SDS and β-mercaptoethanol and boiled for 10 min, which caused the fibrillar BSA to disassemble and migrate to the same position as G-BSA on SDS-PAGE.

**Figure 2:**
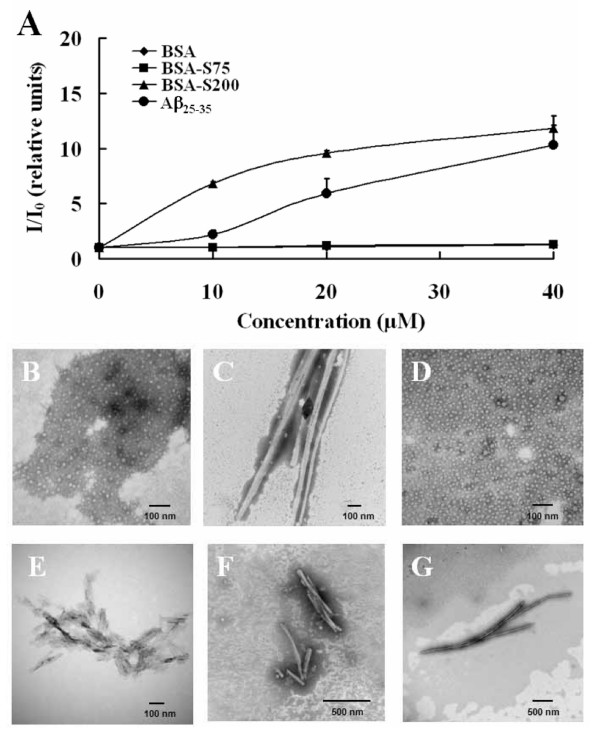
**Analysis of fibrillar BSA by using ThT and transmission electron microscopy**. (A) Comparison of fluorescence levels of increasing concentrations of G-BSA, BSA-S200, BSA-S75 and Aβ_25–35 _after incubating with 20 μM amyloid-specific dye ThT for 1 h. Amyloid Aβ_25–35 _that had been dissolved in sterile double-distilled water and aged at 37°C for 3 days was used as a positive control. The fluorescence level of G-BSA was similar to that of BSA-S75. Data are means ± S.D. (n = 3). TEM images show structure of G-BSA (B), BSA-S200 (C), BSA-S75 (D), Aβ_25–35 _(E), BSA-HW55S (F) and BSA-Zwit (G).

Under certain extreme conditions such as high temperature, glycation, or sonication, proteins can be refolded and converted into fibrils [1-7]. In this study, BSA was refolded by being dissolved in 1% SDS solution, passed through the gel filtration column Superdex-200 and eluted with buffer solution containing 25 mM Tris-HCl, pH 8.0, 1 mM EDTA, 0.1 M NaCl, and 0.05% SDS (Fig. 1). The refolded BSA protein obtained from the Superdex-200 column (BSA-S200), like amyloid fibrils, exhibited enhanced fluorescence level of amyloid-specific dye ThT in a dose-dependent manner (Fig. 2A), as compared with BSA not processed by the Superdex-200 column. These results were substantiated by transmission electron microscopy (TEM) analysis showing BSA with a globular structure (Fig. 2B) and BSA-S200 with a fibril structure (Fig. 2C).

To investigate the effect of gel-filtration column pore size on the formation of fibrils, BSA was eluted with same buffer through a gel filtration column with a smaller pore size (Superdex-75) than that of the Superdex-200 column. TEM analysis revealed that BSA eluted from Superdex-75 (BSA-S75), like BSA, showed a globular structure (Fig. 2D) and no enhanced ThT fluorescence (Fig. 2A). The effect of column matrix on fibrillar protein formation was further examined with a HW55S gel filtration column with pore size similar to that of Superdex-200 but a different matrix composite. BSA eluted from HW55S chromatography (BSA-HW55S), like BSA-S200, displayed a fibrillar structure (Fig. 2F). These data suggest that a molecular sizing column such as Superdex-200 (S200) or HW55S with a pore size more than 70 kDa promotes the formation of amyloid-like fibrillar proteins in the presence of a low concentration of SDS detergent.

We also investigated whether other detergents have the same effect as SDS. BSA solution in the presence of 1% Zwittergent 3–14, which retains its zwitterionic character over a wide range of pH, was eluted from a Superdex-200 column with a buffer solution containing 25 mM Tris-HCl, pH 8.0, 1 mM EDTA, 0.1 M NaCl, and 0.05% Zwittergent 3–14. TEM revealed the BSA protein obtained from a Superdex-200 column with Zwittergent 3–14 (BSA-Zwit), like BSA-S200, with a fibrillar structure (Fig. 2G). These data suggest that not only anionic detergent(s) but also zwitterionic detergent(s) are effective in facilitating fibrillar protein formation.

### Fibrillar BSA induces apoptosis in BHK-21 cells

**Figure 3:**
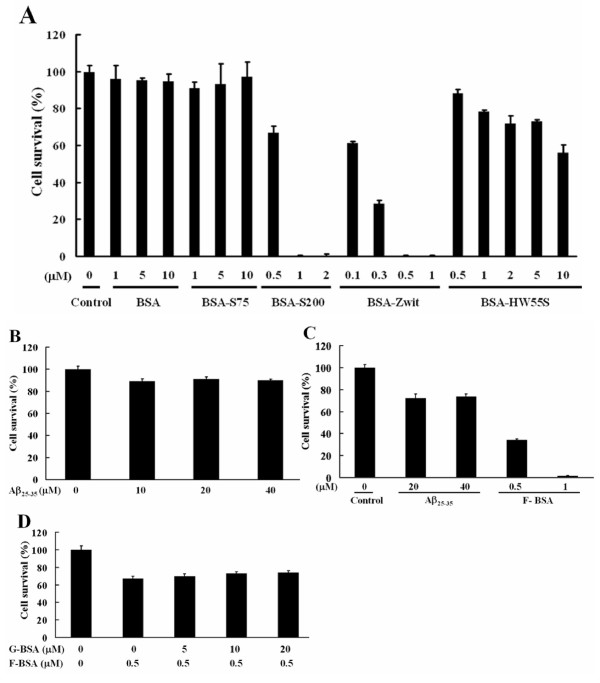
**Cytotoxic effect of Fibrillar BSAs**. Cell viability was determined by the MTT assay. (A) BHK-21 cells were treated for 8 h with various concentrations of G-BSA, BSA-S75, BSA-S200, BSA-Zwit, or BSA-HW55S as indicated in serum-free medium. (B) BHK-21 cells were treated with increasing concentrations of Aβ_25–35 _in serum-free medium for 8 h. (C) BHK-21 cells were treated with increasing concentrations of Aβ_25–35 _or F-BSA (BSA-S200) in serum-free medium for 24 h. (D) BHK-21 cells were treated with or without increasing concentrations of G-BSA (BSA) for 1 h, then incubated with 0.5 μM F-BSA (BSA-S200) in serum-free medium for 8 h. Data are means ± S.D. (n = 3) of percentage of cell survival as determined by the MTT assays.

**Figure 4:**
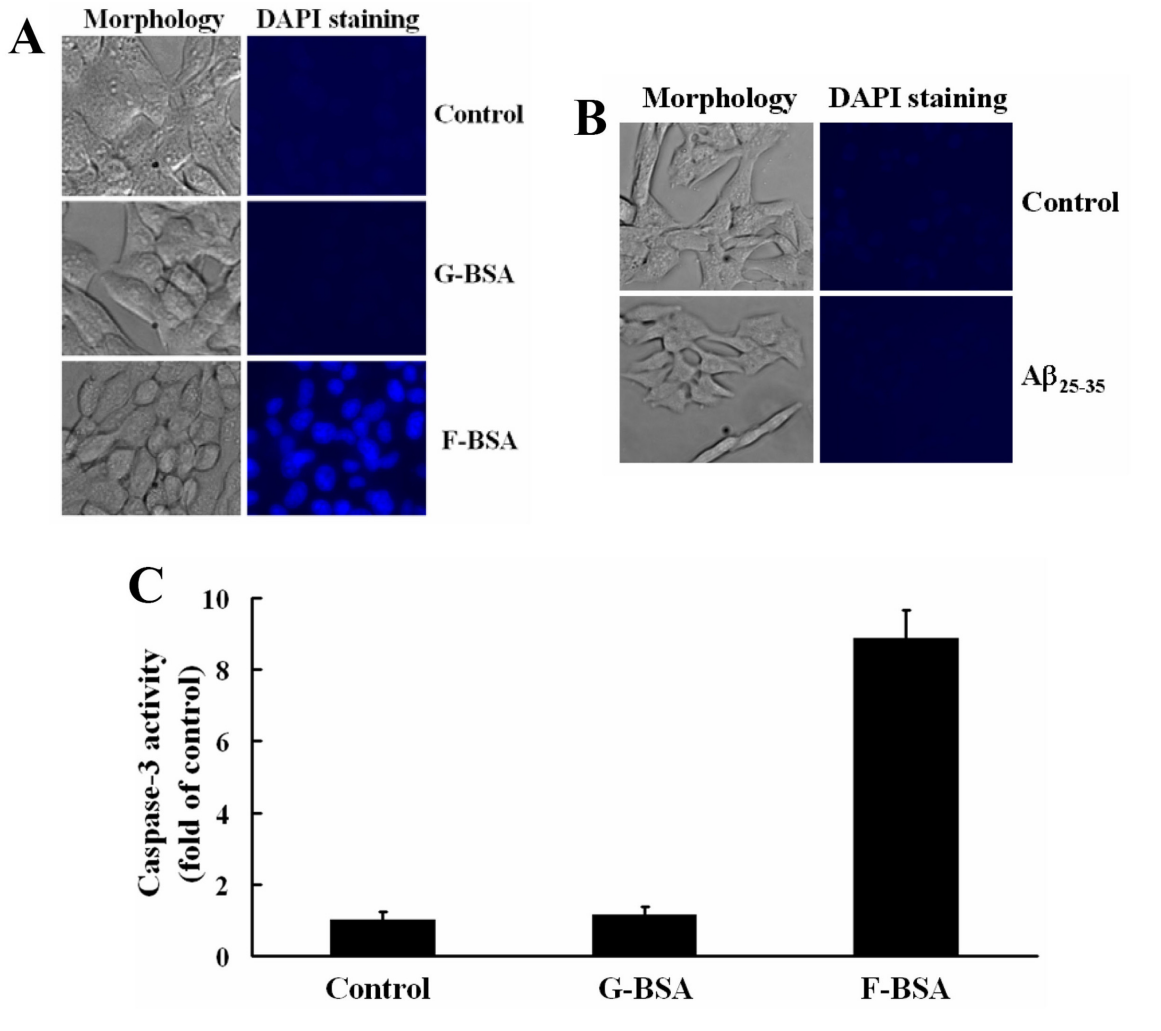
**Evaluation of the apoptotic effect of fibrillar BSA**. (A) BHK-21 cells were incubated with 1 μM G-BSA (BSA) or F-BSA (BSA-S200) for 3 h. The cells were observed under a fluorescence microscope, and their nuclei were stained with DAPI (magnification in all panels, ×400). (B) BHK-21 cells were incubated with 40 μM Aβ_25–35 _for 3 h. The cells were observed under a fluorescence microscope, and their nuclei were stained with DAPI (magnification in all panels, ×400). (C). BHK-21 cells were cultured with 0.8 μM G-BSA (BSA) or F-BSA (BSA-S200) for 15 h in serum-free medium, then underwent caspase-3 activity analysis measured by fluorogenic substrate as described under "Methods". Data are the mean ± SD of three experiments.

Since amyloid-like fibrils are cytotoxic to neuronal cells [20-23], we examined whether fibrillar BSAs induced by our method are cytotoxic to cells, we treated BHK-21 cells with various concentrations of BSA-S200, BSA-Zwit, or BSA-HW55S in serum-free medium for 8 h. BSA-S200, BSA-Zwit, and BSA-HW55S were all cytotoxic to cells in a dose-dependent manner (Fig. 3A). BSA-Zwit exhibited the strongest cytotoxicity among all tested. At 0.5 μM concentration, it induced almost 100% cytotoxicity, whereas BSA-S200 induced 35% and BSA-HW55S 10% cytotoxicity. The IC_50 _for BSA-Zwit, BSA-S200 and BSA-HW55S was 0.2, 0.75 and more than 10 μM, respectively. As controls, two globular proteins, BSA and BSA-S75 induced little, if any, cytotoxicity to cells (Fig. 3A). Interestingly, the cytotoxicity induced by all fibril BSAs (F-BSAs) in BHK-21 cells was stronger than amyloid Aβ_25–35 _which induced a mere 10% cytotoxicity at concentrations as high as 40 μM after 8 h incubation (Fig. 3B). Significant number of cell death induced by Aβ_25–35 _could be observed after 24 h incubation (Fig. 3C). Pre-treating BHK-21 cells with increasing concentrations of globular BSA (G-BSA) did not reverse the cytotoxicity induced by BSA-S200 (Fig. 3D). To examine whether F-BSA-induced cytotoxicity was related to cellular apoptosis, DAPI staining and measurement of caspase-3 activity were performed. Our results revealed that F-BSA induced nuclei condensation (Fig. 4A) and increased caspase-3 activity (Fig. 4C) as compared with G-BSA and Aβ_25–35 _(Fig. 4B). Taken together, these results suggest that treatment with F-BSA induces cytotoxicity and apoptosis of cells and this effect of F-BSA is not reversed by treatment with G-BSA.

### Fibrillar BSA induces apoptosis via integrin/FAK/Akt/GSK-3β pathway

**Figure 5:**
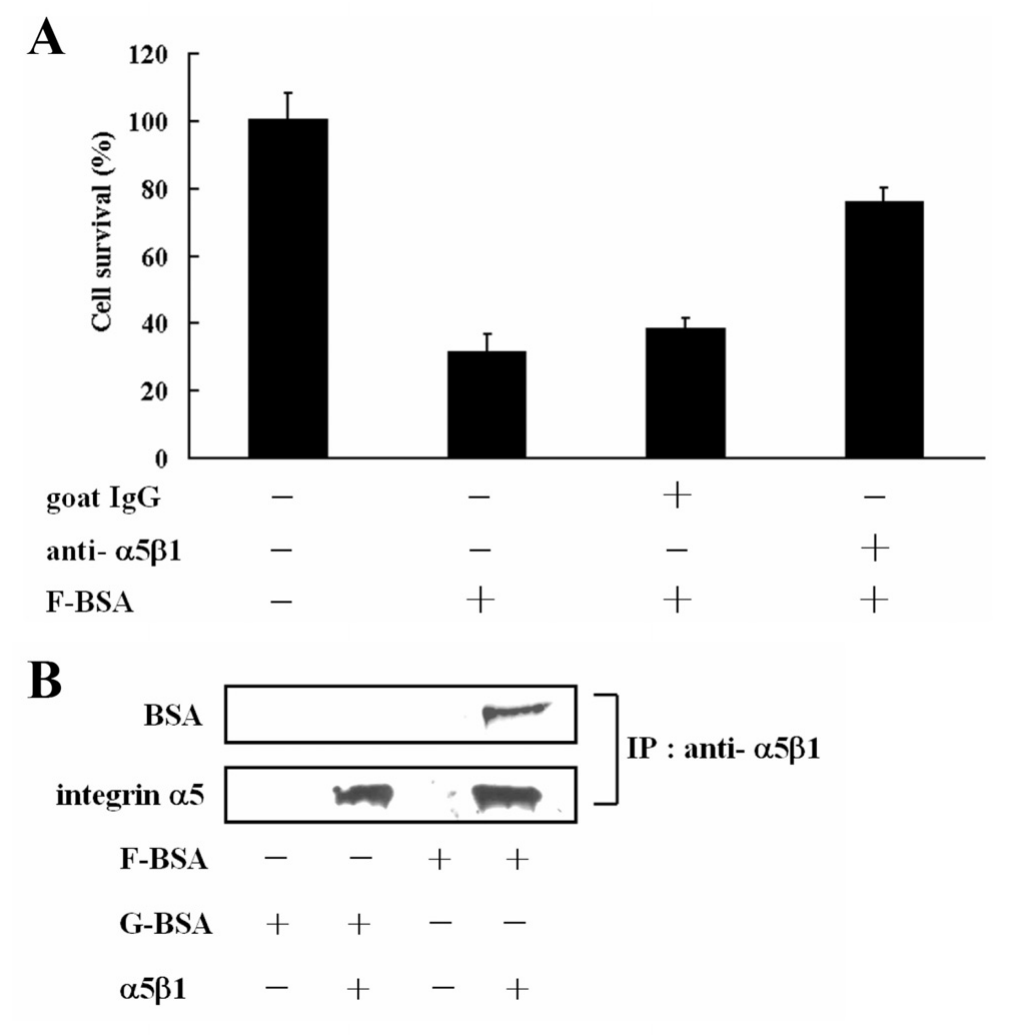
**Interaction between fibrillar BSA and integrin α5β1**. (A) T47D cell lines were pre-treated with or without 0.67 μM goat IgG or 0.67 μM goat anti-integrin α5β1 antibody for 30 min as indicated, then incubated with 2 μM F-BSA (BSA-S200) in serum-free medium for 8 h. Cell viability was determined by the MTT assay. Data are means ± S.D. (n = 3). (B) Integrin α5β1 protein was linked to protein A/G beads by use of anti-integrin α5β1 antibody, then incubated with F-BSA (BSA-S200) or G-BSA (BSA) overnight. The immunocomplexes were separated by SDS-PAGE and immunoblotted with anti-integrin α5 and anti-BSA antibodies.

Being cytotoxic to BHK-21 cells, F-BSA was also cytotoxic to cancer cells such as T47D cells (a breast cancer line) (Fig. 5A). To examine whether the apoptotic effects of F-BSA is via integrins known to modulate various processes such as cell proliferation, morphology, migration, and apoptosis [37-41], T47D cells were pre-treated with anti-integrin α5β1 antibody for 30 min, then incubated with F-BSA (e.g., BSA-S200) for 8 h in serum-free medium. Cell viability results revealed that pre-treating T47D cells with anti-integrin α5β1 antibody but not goat IgG diminished the cytotoxic effect of F-BSA (Fig. 5A). The interaction between F-BSA and integrin was further verified by immunoprecipitation. Incubation of control beads or integrin α5β1 protein-linked beads with G-BSA or F-BSA revealed that F-BSA but not G-BSA bound to integrin α5β1 (Fig. 5B).

**Figure 6:**
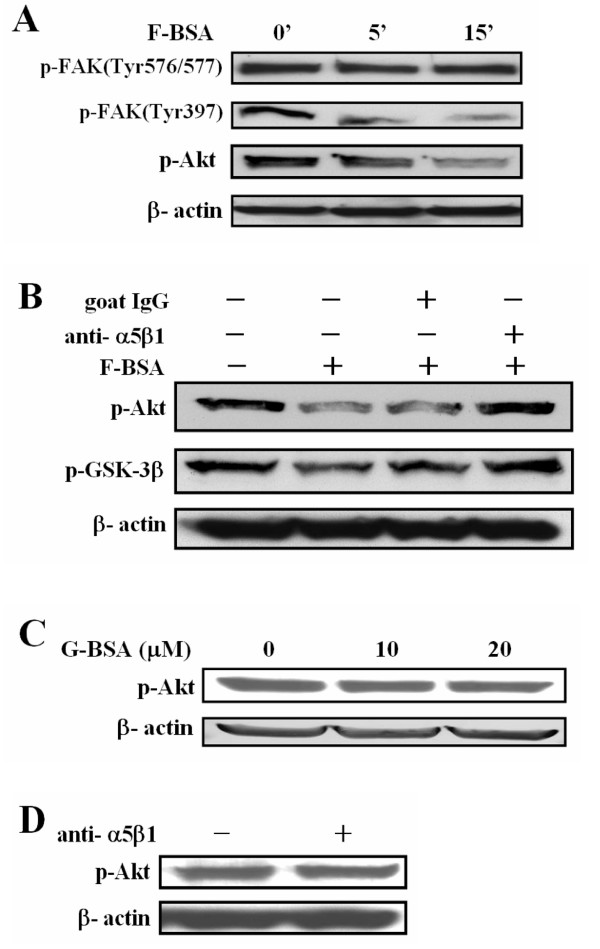
**Fibrillar BSA induced cytotoxicity via the integrin/FAK/Akt pathway**. (A) BHK-21 cells were treated with 3 μM F-BSA (BSA-S200) in serum-free medium for the indicated time, and cell lysates were analyzed by western blotting with anti-phospho-FAK(Tyr576/577), anti-phospho-FAK(Tyr397), and anti-phospho-Akt (p-Akt) antibodies. (B) BHK-21 cells were pre-treated for 30 min with or without 1 μM goat IgG or 1 μM goat anti-integrin α5β1 antibody as indicated, then treated with 3 μM F-BSA (BSA-S200) in serum-free medium for 15 min. Cell lysates were analyzed by western blotting with anti-phospho-Akt (p-Akt) and anti-phospho-GSK-3β (p-GSK-3β) antibodies. (C) BHK-21 cells were treated with increasing concentrations of G-BSA (BSA) in serum-free medium as indicated, and cell lysates were analyzed by western blotting with anti-phospho-Akt (p-Akt) antibody. (D) BHK-21 cells were treated with or without 1 μM anti-integrin α5β1 antibody in serum-free medium for 30 min, and cell lysates were analyzed by western blotting with anti-phospho-Akt (p-Akt) antibody.

We then investigated whether the molecules involved in the integrin signaling-pathway cascade, such as FAK, Akt and GSK-3β, are affected by F-BSA. F-BSA dephosphorylated FAK at Tyr 397 but not Tyr 576/577 in a time-dependent manner (Fig. 6A). Western blot analysis revealed that F-BSA dephosphorylated both Akt and GSK-3β (Fig. 6B). The effect of F-BSA on Akt and GSK-3β phosphorylation could be reversed by pre-treating cells with anti-integrin α5β1 antibody (Fig. 6B). In comparison, G-BSA, as well as anti-integrin α5β1 antibody, had no effect on the phosphorylation of Akt (Figs. 6C and 6D). Thus, F-BSA induces apoptosis via an integrin/FAK/Akt/GSK-3β/caspase-3 pathway.

## Discussion

Although proteins usually fold to their native and functional states, under certain conditions, some proteins may fail to fold correctly and form fibrillar products with abnormal biological effects [1-7]. One notable example of incorrectly folded proteins is amyloid aggregate, which is associated with Alzheimer's disease and transmissible spongiform encephalopathy [20-23]. In the present study, we used a simple column chromatography process to induce native proteins, in this case, globular BSA, to undergo fibrillization in the presence of detergent (SDS or Zwittergent 3–14) (Figs. 2 and 3). Our findings are thus consistent with a previous proposal that numerous proteins with diverse structures, including both disease and non-disease associated proteins, are capable of forming fibril amyloid [42].

Aging proteins at 37°C may convert them into amyloid fibrils, but, in most cases, aging requires days to weeks [20-23]. Fibril formation can also be accelerated by SDS [15,16] but still requires vigorous stirring overnight at 37°C with the addition of fibril seeds [15] or 2 days of incubation at room temperature [16]. In addition, unless aggregates form and precipitate out, these methods cannot be used to isolate fibrillar from non-fibrillar proteins. Our column chromatography process converts globular protein into a fibrillar form in an efficient manner and effectively separates the fibril from other forms of the protein. It is thus a novel approach for producing fibrillar proteins.

We show that Superdex-200 and HW55S columns are more efficient than Superdex-75 columns in converting proteins into fibrillar forms. Even though Superdex-200 and HW55S have different matrix composite (cross-linked agarose and dextran vs. hydroxylated methacrylic polymer), they have similar pore size (10–600 vs. 1–700 kDa), which is larger than that of Superdex-75 (3–70 kDa). Although BSA-S200 was more potent than BSA-HW55S (Fig. 3A), these findings seem to suggest, nonetheless, that an appropriate bead pore size plays a crucial role in the column-induced fibril formation, because fibrillization was less with Superdex-75. A likely explanation is that the proteins are reshaped to fibrillar form after entering the pores and passing through the long channel inside the column beads. Alternatively, the reshaping may be a consequence of the greater overall stability resulting from factors such as an increase in van der Waals interactions in the column bead. The explanation remains to be verified by further studies.

Pertinhez et al. found that low concentrations of the anionic detergent SDS promoted fibril formation [16] and proposed that SDS might stimulate aggregation, perhaps by associating with the peptide molecules; once such aggregates are formed, intermolecular β-sheet interactions are likely to develop readily. Several anionic detergents have subsequently been found to be effective for fibrillization [17]. We found that the effect of Zwittergent 3–14 on the formation of cytotoxic BSA was similar to or even better than that of SDS (Fig. 3). TEM showed that there was few, if any, BSA oligomer or protofibril in the eluate of the column chromatography, both BSA-S200 and BSA-Zwit existed in the fibrillar form (Fig. 2C and 2G). The length of BSA-Zwit was in average two fold longer than BSA-S200 (5,000 nm vs. 2,000 nm). As similar concentration of fibrillar BSA-Zwit and BSA-S200 was used in the cytotoxic assay, the difference in potency is likely due to factor(s) other than concentration of amyloid fibrils. We are thus the first to report that zwitterionic detergents are also effective for promoting fibril formation. In addition, Zwittergent 3–14, unlike SDS, contains both anionic and cationic properties, so fibril formation may be stimulated by not only anionic but also cationic properties of the detergents. Further elucidating whether the detergents in this process enhance fibril formation by increasing unfolding or providing stability of the incorrectly refolded proteins, or both, will be interesting.

Although G-BSA is not a ligand for integrin (Fig. 5B), F-BSA caused cellular apoptosis by binding to integrin α5β1 (Figs. 4 and 5). Recently, Wright et al. [29] reported that amyloid-β peptide (Aβ) bound to α2β1 and αVβ1 integrin to activate Pyk2, as well as phosphorylated paxillin and induced neurotoxicity. The authors did not, however, detect any effect in the phosphorylation of FAK [29]. Here, we report that F-BSA mediated cell apoptosis by binding to integrin α5β1, which leads to the dephosphorylation of FAK (Tyr 397), Akt and GSK-3β. The F-BSA produced in this study therefore seemed to deactivate an integrin signaling pathway via a mechanism different from that induced by Aβ. Wright et al. used human cortical neurons, whereas we used BHK-21 and T47D, so whether this difference in mechanism of action is due to the difference in target cells or integrin receptors remains to be clarified.

Aβ and amyloid polypeptides usually undergo multistage assemblies such as oligomers, protofibrils to become fibrils [24-27,43,44]. Even though it is still debatable which state of the polypeptide is more toxic to the cells, the existing data seem to suggest that different assembled macromolecular forms may exert cytotoxicity via different mechanism of actions including membrane perturbation, apoptosis, necrosis etc. [26,27,45-47]. Although fibril proteins have been shown to cause caspase-related apoptosis [43,46,48], the details have not been explored. In this study, we found that fibrillar BSA causes apoptosis by modulating the integrin/FAK/Akt/GSK-3β/caspase-3 signaling pathway.

Because BSA does not contain RGD, a unique binding motif for integrin, it will be interesting to analyze whether the mechanism of binding of fibrillar BSA to integrin is the same as molecules with RGD in their sequence such as vitronectin. Of note, even though some of the RGD-containing peptides are cytotoxic, others, such as fibronectin, are not [49]. In comparison, almost all fibrillar proteins have been documented to cause cytotoxicity [20-23]. Thus, elucidating whether the mechanism of action of all fibrillar proteins in causing cytotoxicity is due to the involvement of a shared structural motif will be of interest.

Detergents and column chromatography have routinely been used to obtain membrane proteins close to their native status [50]. However, whether the isolated membrane proteins are cytotoxic and/or apoptotic has not been reported. Our process could be used to isolate a few membrane proteins to elucidate whether they are fibrillar and apoptotic. If the membrane proteins isolated in this manner do cause apoptosis, then the structure analysis of membrane proteins isolated with the assistance of detergents particularly those with SDS and zwittergents might require re-examination.

## Conclusion

We report on a novel process to convert globular proteins such as BSA into fibrillar form. Unlike globular BSA, which is non-cytotoxic, fibrillar BSA caused cell death by modulating the integrin/FAK/Akt/GSK-3β/caspase-3 signaling pathway. These findings may be useful for improving our understanding of the pathogenesis of amyloid-like fibrils and applicable for the development of better therapeutics that targets the underlying mechanism(s) of the etiologic agents.

## Methods

### Materials

The antibodies against phospho-Try^576/577 ^FAK, phospho-Ser^473 ^Akt, and phospho-Ser^9 ^GSK-3β were purchased from Cell Signaling Technology (Beverly, MA, USA). The antibody against phospho-Tyr^397 ^FAK was obtained from Biosource (Camirillo, CA, USA). Zwittergent 3–14 was purchased from Calbiochem (San Diego, CA, USA). Integrin α5β1 protein, anti-β-actin antibody, anti-integrin α5 antibody, anti-integrin α5β1 antibody (function-blocking antibody), goat IgG, horseradish peroxidase-coupled anti-mouse IgG secondary antibodies, horseradish peroxidase-coupled anti-rabbit IgG secondary antibodies, and MTT assay kit were purchased from Chemicon (Temecula, CA, USA). Anti-BSA antibody was obtained from Molecular Probes (Eugene, OR, USA). BSA was purchased from Bio Basic Inc. (Markham, Ontario, Canada). Aβ_25–35_, purchased from Sigma (St. Louis, MO, USA), was dissolved in sterile double-distilled water and aged at 37°C for 3 days before use. Thioflavin T (ThT), sodium dodecyl sulfate (SDS), 4', 6' – Diamidino-2-phenylindole dilactate (DAPI), and other chemicals not otherwise specified were obtained from Sigma. Superdex-200 and Superdex-75 beads were obtained from Amersham Biosciences (Uppsala, Sweden), HW55S gel filtration bead was obtained from TOSOH Corporation (Shiba, Tokyo, Japan).

### Preparation of fibrillar BSAs (F-BSAs)

Twenty milligrams of BSA was dissolved in 10 ml of PBS with 1% SDS (w/v). The BSA solution was sonicated for 5 min and subsequently applied to a Superdex-200 or a HW55S column (2.6 × 100 cm), which was previously equilibrated with the eluting solution (25 mM Tris-HCl, pH 8.0, 1 mM EDTA, 0.1 M NaCl, and 0.05% SDS). The column was eluted at the rate of 1 ml/min and fractions 28 – 32 that contained BSA were pooled. The pooled fractions were then dialyzed against PBS to remove SDS. The yield of the fibrillar BSA was about 67%. TEM showed that the eluate of Sephadex-200 contained fibrillar BSA which was not increased or decreased by the dialysis process (data not shown).

### Transmission electron microscopy (TEM)

For TEM analysis of fibrillar proteins, 1 mg/ml of protein was applied to a 200-mesh carbon-coated copper grid. Excess samples were removed, and the grid was air dried. The protein-bearing grid was negatively stained with 1% (W/V) phosphotungstic acid for 1 min. Transmission electron micrographs were observed at 20,000–150,000× magnification at 75 kV on a Hitachi H-7000 electron microscope.

### Thioflavin T (ThT) fluorescence

Binding to ThT is one of the characteristics of amyloid-like proteins [10]. For fluorescence measurements, increasing concentrations of proteins (10 μM, 20 μM, and 40 μM) were incubated with 20 μM ThT. After 1 h of incubation at room temperature, fluorescence was measured in triplicate on a Wallac VICTOR^2 ^1420 Multilabel Counter (Perkin Elmer Life Science, Waltham, USA). Excitation and emission wavelengths were 430 nm and 486 nm, respectively. ThT background signal from buffer was subtracted from corresponding measurements.

### Cell lines and treatments

BHK-21 cells (baby hamster kidney; ATCC CRL-1632) and T47D cells (human breast duct carcinoma; ATCC HTB-133) were maintained at 37°C in Dulbecco's modified Eagle's medium (DMEM) supplemented with 10% fetal bovine serum (FBS), 2 mM L-glutamine, 100 units/ml penicillin, and 100 μg/ml streptomycin. In brief, cells were seeded 24 h prior to treatment. The cells were washed twice with PBS and incubated with proteins in serum-free DMEM for the indicated time. Cells were then lysed with 0.2 ml lysis buffer (Pierce, Rockford, USA) at the indicated time points, and 30 μg of cell lysates were analyzed for FAK, Akt, and GSK-3β phosphorylation by western blot analysis.

### Assay for cell survival and apoptosis

Cell survival was determined by MTT colorimetric assay. Exponentially growing cells (1 × 10^4 ^cells/well for BHK-21 cells; 1.25 × 10^4 ^cells/well for T47D cells) were seeded in 96-well plates in DMEM with 10% FBS and incubated for 24 h. Treatment of cells with a series of concentrations of proteins was carried out in serum-free DMEM for 8 h at 37°C. After treatment, MTT solution was added to each well (0.5 mg/ml), followed by 4 h incubation. The viable cell number is directly proportional to the production of formazan, which, following solubilization with isopropanol, can be measured spectrophotometrically at 560 nm by an ELISA plate reader. To visualize DNA condensation in nuclei, the control and treated cells were incubated in serum-free DMEM for 3 h and stained with DAPI (0.5 μg/ml in PBS) according to the manufacturer's instructions and then examined under fluorescence microscope.

### SDS-PAGE and immunoblot analyses

Cell lysates were resolved by 10% SDS-PAGE in Hoefer vertical gel apparatuses (Amersham Biosciences), followed by electrophoretic transfer to polyvinylidene difluoride membranes. The membranes were blocked with 5% skim milk powder in 5 mM Tris-HCl, pH 7.4, 136 mM NaCl, 0.1% Tween-20 (TBST buffer) for 1 h and incubated with primary antibody (5–10 μg/ml) in blocking buffer. The membranes were washed in TBST, and then incubated with horseradish peroxidase-conjugated secondary antibody. The proteins were detected on a Biomax ML film using the supersignal west pico chemiluminescent substrate kit (Pierce).

### Immunoprecipitation assay

Integrin α5β1 protein was incubated with anti-integrin α5β1 antibody (200 μg/ml) and protein A/G beads (Santa Cruz Biotechnology, Santa Cruz, USA) at room temperature for 2 h. The resulting beads were then incubated with globular BSA (G-BSA) or fibrillar BSA (F-BSA) overnight at 4°C. The immunocomplexes were collected by centrifugation and the pellet was washed with PBS. After three washes, recovered immunocomplexes were solubilized in sample buffer solution containing 1% SDS and β-mercaptoethanol, boiled for 10 min, and separated by 10% SDS-PAGE. The proteins were revealed by immunoblotting with anti-integrin α5 and anti-BSA antibodies.

### Caspase-3 activity assay

Caspase-3 activity was determined by the cleavage of the fluorometric substrate z-DEVD-AMC (Upstate Biotechnology, Lake Placid, USA) according to the manufacturer's instructions. In brief, cells were harvested and washed twice in PBS, and lysed in a lysis buffer (Pierce) supplemented with protease inhibitor mixture (Sigma). The lysates underwent centrifugation at 12,000 × *g *for 15 min at 4°C, and protein concentrations in the supernatants were determined by use of Bio-Rad Protein Assay. An amount of 50 μg of the cell lysates were incubated with 72 μM z-DEVD-AMC at room temperature for 15 min in triplicate. Cleavage of z-DEVD-AMC was determined by measurement of emission at 460 nm after excitation at 380 nm with the fluorescence plate reader.

## Authors' contributions

CYH carried out the preparation of fibrillar BSA, analyzed the fibrillar BSA-induced signaling pathway, and drafted the manuscript. CLC carried out the preparation of fibrillar BSA and assayed the cytotoxicity of fibrillar BSA. CML participated in the design of the study and helped to draft the manuscript. SML conceived of the study, participated in its design and coordination and finalized the manuscript. All authors read and approved the final manuscript.
